# Ipilimumab with temozolomide vs. temozolomide alone after surgery and chemoradiotherapy in recently diagnosed glioblastoma: a randomized phase II clinical trial

**DOI:** 10.1093/noajnl/vdaf032

**Published:** 2025-05-26

**Authors:** Nicholas F Brown, Catherine McBain, Lucy Brazil, Sharon Peoples, Sarah Jefferies, Fiona Harris, Anup Vinayan, Puneet Plaha, Claire Brooks, Samia Hussain, Susan J Dutton, Jonathan Cook, Stasya M Ng, Stephanie Levy, Timothy Coutts, Paul Mulholland

**Affiliations:** Department of Medical Oncology, University College London Hospitals, London, UK; Department of Clinical Oncology, The Christine NHS Foundation Trust, Manchester, UK; Department of Clinical Oncology, Guys and St Thomas’ NHS Foundation Trust, London, UK; Edinburgh Centre for Neuro-Oncology, Western General Hospital Edinburgh Cancer Centre, Edinburgh, UK; Department of Clinical Oncology, Cambridge University Hospitals NHS Foundation Trust, Cambridge, UK; Department of Clinical Oncology, Cambridge University Hospitals NHS Foundation Trust, Cambridge, UK; Department of Clinical Oncology, Mount Vernon Cancer Centre, Northwood, UK; Nuffield Department of Surgical Sciences, Oxford University Hospitals, Oxford, UK; Oncology Clinical Trials Office (OCTO), Department of Oncology, The University of Oxford, Oxford, UK; Oxford Clinical Trials Research Unit, Centre for Statistics in Medicine (CSM), University of Oxford, Botnar Research Centre, Oxford, UK; Oxford Clinical Trials Research Unit, Centre for Statistics in Medicine (CSM), University of Oxford, Botnar Research Centre, Oxford, UK; Oxford Clinical Trials Research Unit, Centre for Statistics in Medicine (CSM), University of Oxford, Botnar Research Centre, Oxford, UK; Oncology Clinical Trials Office (OCTO), Department of Oncology, The University of Oxford, Oxford, UK; Oncology Clinical Trials Office (OCTO), Department of Oncology, The University of Oxford, Oxford, UK; Oncology Clinical Trials Office (OCTO), Department of Oncology, The University of Oxford, Oxford, UK; Glioblastoma Research Group, UCL Cancer Institute, London, UK; Department of Medical Oncology, University College London Hospitals, London, UK

**Keywords:** glioblastoma, ipilimumab, immunotherapy, checkpoint inhibitor, clinical trial

## Abstract

**Background:**

Glioblastoma confers a bleak prognosis, with median survival of less than a year. This trial evaluated whether addition of the CTLA-4 immune checkpoint inhibitor ipilimumab to standard therapy improves survival in patients with recently diagnosed glioblastoma.

**Methods:**

Ipi-Glio was a stratified randomized, open-label, multicenter, academic phase II study. Patients with recently diagnosed de novo glioblastoma following completion of chemoradiotherapy were randomized 2:1 to ipilimumab + temozolomide (Arm A) vs temozolomide alone (Arm B), stratified to extent of surgery and MGMT promotor methylation. Primary endpoint was overall survival. Secondary endpoints included progression-free survival at 18 months, overall survival at 3 years, and toxicity (≥Grade 3).

**Results:**

One hundred nineteen patients were randomized (79 to Arm A, 40 to Arm B). Patient characteristics (Arm A vs B): median age 57 vs 49 years; male sex 70 vs 65%, gross total resection 61 vs 60%, tumor MGMT promotor methylation 39 vs 40%. Median overall survival was 18 months (60% CI 16.0, 23.9) in Arm A vs 23.0 months (17.3, 26.4) in Arm B (adjusted HR 1.09, 60% CI 0.86,1.38, one-sided *P* = .62; logrank *P* = .75). Progression-Free Survival: 10.8 vs 12.5 months (Arm A vs B) (adjusted HR 1.34, 1.06–1.68, one-sided *P* = .86;logrank *P* = .42). Grade 3 or above adverse events: 53% Arm A vs 43% Arm B (*P* = .27).

**Conclusions:**

No benefit was observed with the addition of ipilimumab to temozolomide in patients with recently diagnosed glioblastoma following chemoradiotherapy. This study does not support further investigation of this regimen in this setting.

**Trial Registration:**

ISRCTN84434175 (www.isrctn.com/ISRCTN84434175)

**Key Points**

- This study investigated adding ipilimumab to standard therapy in recently diagnosed glioblastoma.
- Patients were randomized after radiotherapy to temozolomide ± ipilimumab.
- No progression-free or overall survival benefit was found with the addition of ipilimumab.

**Importance of the Study**

Immune checkpoint inhibitors are being used in an expanding range of cancer tumor types. Whilst CTLA-4 inhibition is effective in preclinical models of glioblastoma, the efficacy of CTLA-4 inhibition in patients with glioblastoma remains unknown. This is the first clinical trial to evaluate whether the addition of the CTLA-4 immune checkpoint inhibitor ipilimumab to standard therapy improves survival in patients with glioblastoma. In this phase 2 multicenter study, patients with recently diagnosed de novo glioblastoma following completion of chemoradiotherapy were randomized to ipilimumab with temozolomide vs temozolomide alone. Unfortunately, no progression free or overall survival benefit was found with the addition of ipilimumab. This study does not support further exploration of ipilimumab with temozolomide following chemoradiotherapy in glioblastoma.

Glioblastoma is the most common malignant primary brain tumor.^1^ Survival is dismal, with median survival of 6–10 months in real-world cohorts,^2–5^ and 14.6–21.2 months with standard therapy in clinical trials.^6–12^ Standard therapy is surgical debulking if feasible, with adjuvant external beam chemoradiotherapy administered within 6 weeks of surgery, comprising 60 Gray (Gy) in 30 fractions given over 6 weeks along with daily concomitant temozolomide 75 mg/m^2^. After a 28-day break, patients receive 6 cycles of adjuvant temozolomide 150–200 mg/m^2^, given for 5 days in a 28-day cycle. This standard was approved in 2005 following demonstration of a 2.5-month medial survival benefit over radiotherapy alone.^10,13^ Patients with a gross total resection have better survival than those with subtotal resection in meta-analyses of retrospective cohort studies.^14–17^ Patients with epigenetic silencing of the DNA repair enzyme MGMT through methylation of the promotor region have both improved survival and improved response to temozolomide, established in both prospective randomized clinical trials and real-world retrospective cohorts.^18,19^

Glioblastomas elicit dysregulation of the systemic immune system. Meanwhile the CNS, whilst traditionally considered immune-privileged, is now recognized to fully interact with the innate and adaptive immune system, albeit in a tightly regulated manner.^20^ The amplitude and quality of T-cell responses against an antigen are regulated by a balance of co-inhibitory and co-stimulatory signals termed immune checkpoints. Gliomas exploit these checkpoints with expression of negative immune regulators in order to escape immune surveillance.^20,21^

Ipilimumab is a fully human monoclonal antibody targeting the Cytotoxic T-lymphocyte antigen-4 (CTLA-4) inhibitory immune checkpoint.^22^ Systemic CTLA-4 blockade prolongs survival in preclinical glioblastoma models.^23,24^ Efficacy in brain metastases in patients with melanoma provides clinical evidence within the central nervous system.^25,26^

The aim of this randomized phase II trial was to investigate the addition of ipilimumab to standard therapy in patients with recently diagnosed glioblastoma.

## Methods

### Study Design and Participants

In this open-label, multicenter, phase II clinical trial patients were randomized 2:1 to receive either ipilimumab with temozolomide or temozolomide alone, with randomization minimized by extent of surgery (total vs subtotal resection) and tumor MGMT promotor methylation (methylated, unmethylated, or unknown) using a central randomization system.

Eligible patients were 18–70 years old with an ECOG performance status of 0-1, with newly diagnosed histologically confirmed de novo supratentorial glioblastoma (2016 WHO Classification of CNS Tumors) who had had >20% tumor surgical debulking, had completed standard radiotherapy with concomitant temozolomide (radiotherapy to have begun within 49 days of surgery) and were deemed clinically appropriate to receive adjuvant temozolomide. Patients were ineligible if they had secondary or multifocal glioblastoma, had received any other treatment for glioblastoma, were on >3 mg dexamethasone daily, had metastatic or leptomeningeal disease, or had a clinically relevant, active, known, or suspected autoimmune disease. Full criteria are available in the study protocol, available in the supplemental attachments.^27^

Patient recruitment was paused during the first 2 waves of the COVID-19 pandemic (March to July 2020, September to October 2020), and thus recruitment was extended. Due to the pandemic, a number of protocol deviations (for example, telephone rather than in-person consultations) were observed; the trial management group determined that these did not impact the quality of data or trial outcomes.

### Study Objectives

The primary objective was to evaluate whether the addition of ipilimumab to the current standard of care will improve survival in patients with newly diagnosed glioblastoma. Secondary objectives were evaluation of the safety and tolerability of ipilimumab with temozolomide, progression-free survival (PFS), and long-term survival outcomes. Accordingly, the primary study endpoint was overall survival (OS), with secondary endpoints of PFS at 18 months, overall survival at 3 years, and any toxicity grade 3 or higher according to CTCAE v4.03. Survival was timed from randomization until death from any cause.

### Procedures

Ipilimumab 3 mg/kg every 3 weeks for 4 doses was administered intravenously over 90 minutes, with the first dose administered within 14 days of completing radiotherapy and within 5 days of randomization. Oral temozolomide 150–200 mg/m^2^ was administered daily for 5 days in a 28-day cycle for 6 cycles as per standard of care, starting approximately 4 weeks after completion of chemoradiotherapy. Patients attended study visits for 52 weeks following randomization. Tumor assessment was assessed by contrast-enhanced MRI performed every 12 weeks, or as per local standard of care. Survival status was collected at 18 months from the date the last patient was randomized, and at 2 and 3 years from each patient’s randomization date.

### Statistical Analyses

Overall survival was defined as the time from randomization to death due to any cause. Participants who had not died were censored at their last known alive date. PFS was defined as time from randomization until progression (as determined by Response Assessment in Neuro-Oncology criteria or a multidisciplinary team meeting) or death. We planned to recruit 120 patients in order to show a one-sided significant difference at 20% in median survival of 22.5 months in the ipilimumab with temozolomide arm vs 15 months in the temozolomide alone arm, providing 80% power, allowing for 5% loss to follow-up at 3 years, and assuming an 18-month recruitment period and survival follow-up of 18 months. OS and PFS was compared between 2 arms using Cox proportional hazards regression adjusted for the stratifying factors (MGMT promotor methylation status and extent of resection) with hazard ratios (with 60% confidence intervals). One-sided *P*-values were generated for these 2 tests, otherwise 2-sided 60% intervals were generated with corresponding 2-sided *P*-values. Logrank tests were also used to compare OS and PFS. No data was imputed.

### Study Oversight

This study was conducted in accordance with the Declaration of Helsinki, the principles of Good Clinical Practice, and applicable clinical trials regulations. Study conduct was approved by the South Central (Oxford B) Research Ethics Committee (18/SC/0525) and the Medicines and Healthcare Regulatory Agency (UK). It was conducted by the Oncology Clinical Trials Office (OCTO) in conjunction with Oxford Clinical Trials Research Unit (OCTRU) at the University of Oxford. Trial Registration: ISRCTN84434175 (www.isrctn.com/ISRCTN84434175).

## Results

**Figure 1.**
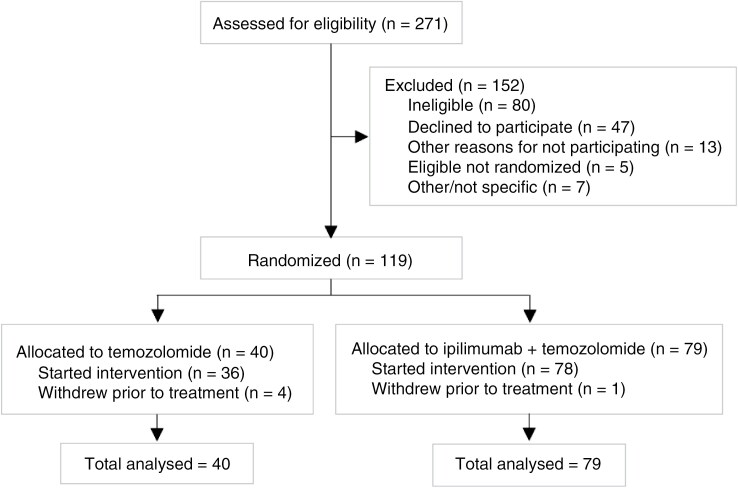
CONSORT diagram

One hundred nineteen patients were randomized (79 to ipilimumab with temozolomide; 40 to temozolomide alone) at 7 sites in the United Kingdom between December 2018 and April 2021. 1 patient in the ipilimumab arm and 4 patients in the temozolomide arm withdrew following randomization but prior to commencing treatment; these patients are included as per intention-to-treat analyses (CONSORT diagram, Figure 1).

Baseline characteristics are displayed in Table 1. Forty-eight (61%) of patients had received a gross total resection in the ipilimumab arm, compared to 24 (60%) in the temozolomide alone arm. Tumor MGMT promotor was methylated in 31 (39%) of patients in the ipilimumab arm vs 16 (40%) of patients in the temozolomide alone arm. Median age was 54 years (ipilimumab arm 57 years vs temozolomide arm 49 years); 81 (68%) of participants were male (55 (70%) ipilimumab arm vs 26 (65%) temozolomide alone); and 83 (70%) had a performance status of 0 (55 (70%) ipilimumab arm vs 28 (70%) temozolomide alone). Seventy-four (63%) of patients completed 6 cycles of temozolomide (48 (61%) ipilimumab arm vs 26 (67%) temozolomide alone) (Supplementary Table 1).

**Table 1.** Baseline Characteristics

	Temozolomide alone *n* = 40	Ipilimumab + Temozolomide *n* = 79	Total *n* = 119
***Median Age, years (IQR)***	49 (40, 56)	57 (48, 61)	54 (44, 60)
***Sex***			
*Female*	14 (35.0%)	24 (30.3%)	38 (31.9%)
*Male*	26 (65.0%)	55 (69.6%)	81 (68.1)
***ECOG performance status***			
*0*	28 (70.0%)	55 (69.6%)	83 (69.8%)
*1*	12 (30.0%)	24 (30.4%)	36 (30.3%)
***Surgical resection status***			
*Gross total resection*	24 (60.0%)	48 (60.7%)	72 (60.5%)
*Subtotal resection*	16 (40.0%)	31 (39.2%)	47 (39.5%)
***MGMT***			
*Methylated*	16 (40.0%)	31 (39.2%)	47 (39.5%)
*Unmethylated*	21 (52.5%)	40 (50.6%)	61 (51.3%)
*Unknown*	3 (7.5%)	8 (10.1%)	11 (9.2%)
***IDH mutation status***			
*Wild-Type IDH*	36 (90.0%)	70 (88.6%)	106 (89.1%)
*IDH 1 mutation*	3 (7.5%)	9 (11.4%)	12 (10.1%)
*IDH 2 mutation*	1 (2.5%)	0 (0.0%)	1 (0.8%)
***Time (median) from surgery to start of radiotherapy*** *, days (IQR)*	35.0 (30.5, 39.0)	35.0 (31.0, 40.0)	35.0 (31.0, 40.0)
***Time (median) from surgery to start of chemotherapy*** *, days (IQR)*	35.0 (31.0, 40.5)	35.0 (31.0, 40.0)	35.0 (31.0, 40.0)

IQR = interquartile range.

**Figure 2.**
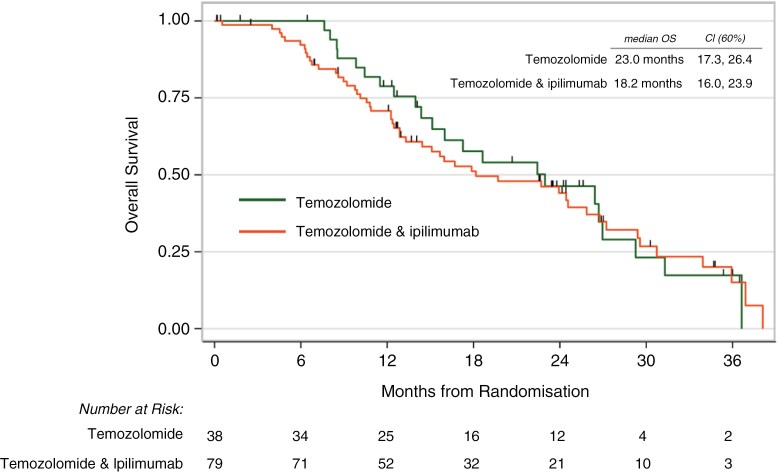
Overall survival (OS) Kaplan–Meier plot

**Figure 3.**
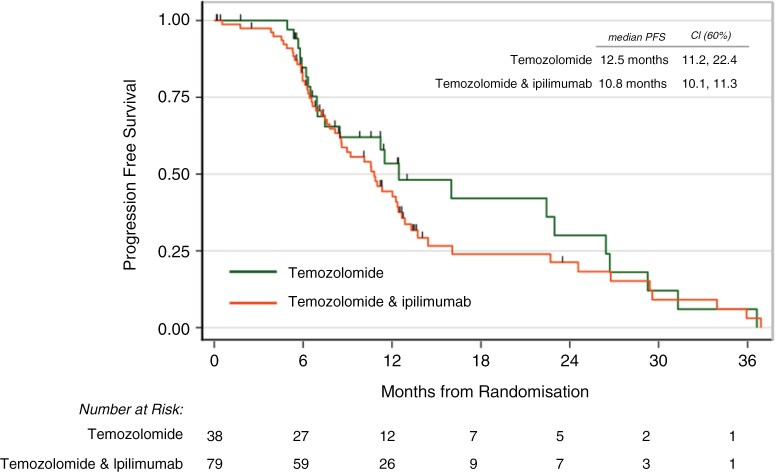
PFS Kaplan–Meier plot

Median OS was 23.0 months (60% CI 17.3, 26.4) in the temozolomide alone group vs 18.2 months (16.0, 23.9) in the ipilimumab group (adjusted HR 1.09, 60% CI 0.86,1.38, one-sided *P* = .62; logrank *P* = .75) (Figure 2). PFS was 12.5 months in the temozolomide along group vs 10.8 months in the ipilimumab group (adjusted HR 1.34, 1.06–1.68, one-sided *P* = .86; logrank *P* = .42) (Figure 3 and Table 2). Tumor MGMT promotor methylation was associated with significantly longer overall survival (HR 0.26, 0.20–0.33 *P* < .001) and PFS (HR 0.44, 0.35–0.55, *P* = .002). Gross total resection was also associated with significantly longer OS (HR 0.40, 0.32–0.51, *P* = .001) and PFS (HR 0.56, 0.44–0.70, *P* = .03) (Supplementary Tables 2 and 3).

**Table 2.** Overall Survival and Progression-Free Survival for Intention to Treat Population

	*Temozolomide alone (n = 40)*		Ipilimumab + temozolomide (n = 79)	
	Survival	60% CI	Survival	60% CI
*OS (median months)*	23.0	17.3, 26.4	18.2	16.0, 23.9
*18 m OS (proportion)*	0.58	0.50, 0.65	0.51	0.46, 0.56
*36 m OS (proportion)*	0.17	0.11, 0.25	0.15	0.10, 0.21
*PFS (median months)*	12.5	11.2, 22.4	10.8	10.1, 11.3
*18 m PFS (proportion)*	0.42	0.33, 0.51	0.24	0.19, 0.29
*36 m PFS (proportion)*	0.06	0.02, 0.12	0.03	0.01, 0.06

Adverse events were reported for 94% of the participants. The median number of adverse events per participant was 6.5 (SD 7.4) in the temozolomide arm vs 10.0 in the ipilimumab arm (SD 10.2). The most common adverse events were fatigue (13 (33) vs 55 (70%)), headache (13 (33) vs 36 (46%)), and nausea (11 (28) vs 31 (39%)) in both the temozolomide alone and the ipilimumab with temozolomide groups respectfully (Supplementary Table 4). There was no significant difference in the number of patients who experienced CTCAE Grade 3 or above adverse events (temozolomide alone 17 (43%) vs 42 (53%) ipilimumab with temozolomide, *P* = .27). Common (≥5% of participants) Grade 3 or above adverse events (Supplementary Table 5) were lymphopenia (8%), vomiting (8%), confusional state (5%), muscular weakness (5%), pulmonary embolism (5%), and thrombocytopenia (5%) in the temozolomide alone group; and seizure (9%), thrombocytopenia (8%), diarrhea (6%), autoimmune colitis (5%), and rash (5%) in the ipilimumab and temozolomide group.

## Discussion

Ipi-Glio is the first clinical trial to evaluate the efficacy of the addition of a CTLA-4 inhibitor to standard therapy in glioblastoma. In this randomized phase II study we investigated the addition of ipilimumab to temozolomide following surgery and chemoradiotherapy in patients with newly diagnosed glioblastoma. No overall survival or PFS benefit was found. The combination of ipilimumab and temozolomide was tolerable, with no new safety signals observed.

Unfortunately, this adds to a growing list of clinical trials that have failed to demonstrate benefit of immune checkpoint inhibition in glioblastoma. CheckMate 498 found no benefit of adjuvant nivolumab with radiotherapy vs temozolomide chemotherapy, and CheckMate 548 found no benefit of adding nivolumab to standard therapy in 2 phase III trials in newly diagnosed glioblastoma in patients with MGMT promotor unmethylated or methylated tumors respectively.^12,28^ Finally, data presented recently from the BN007 phase II/III study of ipilimumab and nivolumab vs temozolomide, given concurrently with radiotherapy then adjuvantly in patients with newly diagnosed glioblastoma found no PFS benefit.^29^ In the relapsed setting, CheckMate 143 found no benefit of nivolumab when compared to bevacizumab.^30^

Yet, reports of patients with radiological responses hint that some patients with glioblastoma do benefit from immune checkpoint inhibitors.^30–33^ Identifying reliable biomarkers to predict response remains the key challenge. The lack of tumor and genomic biomarker analyses that may have identified a subgroup of patients who benefited was a key and regrettable limitation of our study. Several genomic and immune correlates with response to immune checkpoint inhibitors have been proposed.^34–37^ Hope remains that the immune system can be harnessed with targeting of novel immune checkpoints such as IDO-1 or TIM-3, with alternate strategies such as cell therapies, oncolytic viruses or vaccines, or with combination approaches.

Since temozolomide was approved for glioblastoma in 2005, no systemic anticancer agent has demonstrated a survival benefit within a phase III trial. Most phase III trials have either followed promising data from phase II trials compared to historic controls, showed no clear benefit compared to standard therapy, or skipped evaluation in phase II trials entirely.^6,7,12,28,38–40^ Survival in our study was longer than that seen in historic controls and recent trials, with a mOS of over 26 months in the standard therapy arm.^6–12^ This highlights the importance of including standard of care arms within phase II trials, and permits earlier termination of the development of a nonefficacious regimen.

Whilst baseline characteristics were generally well balanced, there was a difference in age between randomized groups, with a younger median age in the temozolomide alone group. Younger age is a recognized positive prognostic marker^2,41–43^ and thus this should be taken into account when interpreting the trial findings. However, we consider it unlikely to have altered the main findings of the study. Further, although patients must not have been on greater than 3 mg daily of dexamethasone at baseline, we did not perform any analysis of dexamethasone use, which is a limitation to interpretation of the study results.

In conclusion, this randomized phase II study found no benefit to the addition of ipilimumab to temozolomide in patients with recently diagnosed glioblastoma following chemoradiotherapy.

## Supplementary Material

vdaf032_suppl_Supplementary_Tables_S1-S5

## Data Availability

The data collected for the study, including individual participant data and a data dictionary defining each field in the set, will be made available to researchers on request to the study team and with appropriate reason, via octo-enquiries@oncology.oc.ac.uk. The shared data will be de-identified participant data and will be available for 5 years following publication of the study. Data will be shared with investigator support, after approval of a proposal and with a signed data access agreement.
