# Supplementary material for: Ipilimumab with temozolomide vs. temozolomide alone after surgery and chemoradiotherapy in recently diagnosed glioblastoma: a randomized phase II clinical trial
Source: Neurooncol Adv. 2025 May 26;7(1):vdaf032. doi: 10.1093/noajnl/vdaf032 (PMC12342986; doi:10.1093/noajnl/vdaf032)
Supplement: vdaf032_suppl_Supplementary_Tables_S1-S5 [file vdaf032_suppl_supplementary_tables_s1-s5.docx]

**Supplemental Table 1: Details of Temozolomide & Ipilimumab cycles completed**

| **Treatment Cycle data (participant level)** | **Temozolomide alone (n=39*)** | | **Ipilimumab+ temozolomide (n=79)** | | **Total (n=119)** | |
| --- | --- | --- | --- | --- | --- | --- |
|  | **n** | **%** | **n** | **%** | **n** | **%** |
| **Temozolomide cycles** |  |  |  |  |  |  |
| No treatment cycles | 4 | 10.3 | 3 | 3.8 | 7 | 5.9 |
| One treatment cycle | 1 | 2.6 | 6 | 7.6 | 7 | 5.9 |
| Two treatment cycles | 2 | 5.1 | 5 | 6.3 | 7 | 5.9 |
| Three treatment cycles | 1 | 2.6 | 6 | 7.6 | 7 | 5.9 |
| Four treatment cycles | 1 | 2.6 | 5 | 6.3 | 6 | 5.0 |
| Five treatment cycle | 4 | 10.3 | 6 | 7.6 | 10 | 8.5 |
| Six treatment cycles | 26 | 66.7 | 48 | 60.8 | 74 | 62.7 |
|  |  |  |  |  |  |  |
| Dose reduction applied | 4 | 11.4 | 10 | 13.2 | 14 | 12.6 |
| **Ipilimumab cycles** |  |  |  |  |  |  |
| No treatment cycles |  | | 1 | 1.3 |  | |
| One treatment cycle |  |  | 4 | 5.1 |  |  |
| Two treatment cycles |  |  | 7 | 8.9 |  |  |
| Three treatment cycles |  |  | 6 | 7.6 |  |  |
| Four treatment cycles |  |  | 61 | 77.2 |  |  |
| Total treatment cycles |  |  | 79 | 100.0 |  |  |

Note: *Cycle data was missing for one participant whom it is known did start their temozolomide treatment.

**Supplemental Table 2: Overall Survival by treatment group**

|  | **Hazard Ratio** |  | **P** | **60% CI** |
| --- | --- | --- | --- | --- |
| **Unadjusted analysis** |  |  |  |  |
| Treatment (unadjusted) | 1.08 |  | 0.62*†* | 0.87, 1.35 |
| **Adjusted analysis*** |  |  |  |  |
| Treatment (adjusted) | 1.09 |  | 0.62*†* | 0.86, 1.38 |
| Methylation status | 0.26 |  | <0.001 | 0.20, 0.33 |
| Cancer resection Status | 0.40 |  | 0.001 | 0.32, 0.51 |

*Notes: * Adjusted for minimisation factors (surgical resection status and methylation status).*

*† One-sided p-value, 20% or 0.20 (favouring the intervention) would be considered significant).*

**Supplemental Table 3: Hazard ratio for Progression-Free Survival by treatment**

|  | **Hazard Ratio** |  | **P** | **60% CI** |
| --- | --- | --- | --- | --- |
| **Unadjusted analysis** |  |  |  |  |
| Treatment (unadjusted) | 1.22 |  | 0.79*†* | 0.99, 1.50 |
| **Adjusted analysis** |  |  |  |  |
| Treatment *(adjusted)* | 1.34 |  | 0.86*†* | 1.06, 1.68 |
| Methylation status | 0.44 |  | 0.002 | 0.35, 0.55 |
| Cancer resection Status | 0.56 |  | 0.03 | 0.44, 0.70 |

*Notes: * Adjusted for minimisation factors (surgical resection status and methylation status).*

*† One-sided p-value, 20% or 0.20 (favouring the intervention) would be considered significant).*

**Supplemental Table 4: Adverse Events (MedDRA preferred term) by treatment group**

| **Adverse Event** | **Temozolomide alone**  ***n (%)*** | **Ipilimumab + Temozolomide**  ***n (%)*** | **Total**  ***n (%)*** |
| --- | --- | --- | --- |
| Abdominal mass | 0 (0) | 1 (1) | 1 (1) |
| Abdominal pain | 1 (3) | 7 (9) | 8 (7) |
| Abdominal pain lower | 0 (0) | 1 (1) | 1 (1) |
| Abdominal pain upper | 0 (0) | 3 (4) | 3 (3) |
| Acne | 0 (0) | 2 (3) | 2 (2) |
| Adrenal insufficiency | 0 (0) | 1 (1) | 1 (1) |
| Alanine aminotransferase increased | 4 (10) | 8 (10) | 12 (10) |
| Alopecia | 6 (15) | 13 (16) | 19 (16) |
| Altered state of consciousness | 0 (0) | 1 (1) | 1 (1) |
| Amnesia | 2 (5) | 0 (0) | 2 (2) |
| Anaemia | 1 (3) | 3 (4) | 4 (3) |
| Anxiety | 2 (5) | 4 (5) | 6 (5) |
| Aphasia | 3 (8) | 13 (16) | 16 (13) |
| Arthralgia | 4 (10) | 9 (11) | 13 (11) |
| Aspartate aminotransferase increased | 1 (3) | 5 (6) | 6 (5) |
| Asthenia | 1 (3) | 2 (3) | 3 (3) |
| Asthma | 0 (0) | 1 (1) | 1 (1) |
| Ataxia | 1 (3) | 0 (0) | 1 (1) |
| Atrial fibrillation | 0 (0) | 1 (1) | 1 (1) |
| Aura | 1 (3) | 0 (0) | 1 (1) |
| Autoimmune colitis | 0 (0) | 4 (5) | 4 (3) |
| Back pain | 2 (5) | 6 (8) | 8 (7) |
| Balance disorder | 1 (3) | 4 (5) | 5 (4) |
| Balanoposthitis | 0 (0) | 1 (1) | 1 (1) |
| Blepharospasm | 0 (0) | 1 (1) | 1 (1) |
| Blood alkaline phosphatase increased | 0 (0) | 2 (3) | 2 (2) |
| Blood bilirubin increased | 0 (0) | 1 (1) | 1 (1) |
| Blood calcium increased | 0 (0) | 1 (1) | 1 (1) |
| Blood cholesterol increased | 0 (0) | 2 (3) | 2 (2) |
| Blood cortisol | 0 (0) | 1 (1) | 1 (1) |
| Blood creatinine increased | 0 (0) | 1 (1) | 1 (1) |
| Blood phosphorus decreased | 0 (0) | 2 (3) | 2 (2) |
| Blood potassium decreased | 0 (0) | 1 (1) | 1 (1) |
| Bradyphrenia | 0 (0) | 1 (1) | 1 (1) |
| Brain oedema | 2 (5) | 2 (3) | 4 (3) |
| Breast mass | 0 (0) | 1 (1) | 1 (1) |
| Catheter site bruise | 0 (0) | 1 (1) | 1 (1) |
| Cellulitis | 0 (0) | 1 (1) | 1 (1) |
| Cerebrospinal fluid leakage | 1 (3) | 0 (0) | 1 (1) |
| Cerumen impaction | 1 (3) | 0 (0) | 1 (1) |
| Chalazion | 0 (0) | 1 (1) | 1 (1) |
| Chest discomfort | 1 (3) | 0 (0) | 1 (1) |
| Chest pain | 1 (3) | 1 (1) | 2 (2) |
| Cognitive disorder | 0 (0) | 2 (3) | 2 (2) |
| Cold-stimulus headache | 0 (0) | 1 (1) | 1 (1) |
| Colitis | 0 (0) | 4 (5) | 4 (3) |
| Communication disorder | 0 (0) | 1 (1) | 1 (1) |
| Confusional state | 2 (5) | 4 (5) | 6 (5) |
| Constipation | 7 (18) | 23 (29) | 30 (25) |
| Contusion | 0 (0) | 2 (3) | 2 (2) |
| Coordination abnormal | 1 (3) | 1 (1) | 2 (2) |
| Cough | 2 (5) | 10 (13) | 12 (10) |
| COVID-19 | 0 (0) | 2 (3) | 2 (2) |
| COVID-19 pneumonia | 1 (3) | 0 (0) | 1 (1) |
| C-reactive protein increased | 0 (0) | 1 (1) | 1 (1) |
| Cushingoid | 4 (10) | 1 (1) | 5 (4) |
| Deafness unilateral | 0 (0) | 1 (1) | 1 (1) |
| Decreased appetite | 4 (10) | 21 (27) | 25 (21) |
| Deep vein thrombosis | 0 (0) | 1 (1) | 1 (1) |
| Dehydration | 1 (3) | 1 (1) | 2 (2) |
| Depressed mood | 1 (3) | 3 (4) | 4 (3) |
| Depression | 0 (0) | 2 (3) | 2 (2) |
| Dermatitis | 0 (0) | 1 (1) | 1 (1) |
| Diarrhoea | 3 (8) | 21 (27) | 24 (20) |
| Diarrhoea haemorrhagic | 0 (0) | 1 (1) | 1 (1) |
| Diplopia | 1 (3) | 1 (1) | 2 (2) |
| Disorientation | 0 (0) | 1 (1) | 1 (1) |
| Dizziness | 2 (5) | 9 (11) | 11 (9) |
| Dizziness postural | 1 (3) | 4 (5) | 5 (4) |
| Dry eye | 1 (3) | 2 (3) | 3 (3) |
| Dry mouth | 0 (0) | 1 (1) | 1 (1) |
| Dry skin | 4 (10) | 10 (13) | 14 (12) |
| Dysaesthesia | 1 (3) | 0 (0) | 1 (1) |
| Dysgeusia | 0 (0) | 1 (1) | 1 (1) |
| Dyspepsia | 2 (5) | 2 (3) | 4 (3) |
| Dysphagia | 1 (3) | 3 (4) | 4 (3) |
| Dyspnoea | 3 (8) | 4 (5) | 7 (6) |
| Ear pain | 1 (3) | 0 (0) | 1 (1) |
| Epistaxis | 0 (0) | 2 (3) | 2 (2) |
| Erectile dysfunction | 0 (0) | 1 (1) | 1 (1) |
| Erythema | 0 (0) | 1 (1) | 1 (1) |
| Eye infection | 1 (3) | 3 (4) | 4 (3) |
| Eye inflammation | 1 (3) | 0 (0) | 1 (1) |
| Eye pain | 1 (3) | 3 (4) | 4 (3) |
| Eye pruritus | 0 (0) | 2 (3) | 2 (2) |
| Face oedema | 1 (3) | 2 (3) | 3 (3) |
| Facial paralysis | 1 (3) | 0 (0) | 1 (1) |
| Facial paresis | 0 (0) | 1 (1) | 1 (1) |
| Fall | 1 (3) | 1 (1) | 2 (2) |
| Fatigue | 13 (33) | 55 (70) | 68 (57) |
| Feeling abnormal | 0 (0) | 2 (3) | 2 (2) |
| Feeling cold | 1 (3) | 0 (0) | 1 (1) |
| Fever | 2 (5) | 5 (6) | 7 (6) |
| Flank pain | 0 (0) | 2 (3) | 2 (2) |
| Flatulence | 0 (0) | 1 (1) | 1 (1) |
| Fungal infection | 0 (0) | 1 (1) | 1 (1) |
| Fungal skin infection | 0 (0) | 1 (1) | 1 (1) |
| Gait disturbance | 0 (0) | 1 (1) | 1 (1) |
| Gastritis | 0 (0) | 2 (3) | 2 (2) |
| Gastrooesophageal reflux disease | 0 (0) | 3 (4) | 3 (3) |
| Generalised tonic-clonic seizure | 1 (3) | 0 (0) | 1 (1) |
| Genital ulceration | 0 (0) | 1 (1) | 1 (1) |
| Gingival bleeding | 0 (0) | 1 (1) | 1 (1) |
| Gout | 0 (0) | 1 (1) | 1 (1) |
| Haematochezia | 0 (0) | 1 (1) | 1 (1) |
| Haematoma | 1 (3) | 0 (0) | 1 (1) |
| Hallucination, olfactory | 0 (0) | 2 (3) | 2 (2) |
| Hallucination, visual | 1 (3) | 0 (0) | 1 (1) |
| Head discomfort | 0 (0) | 3 (4) | 3 (3) |
| Headache | 13 (33) | 36 (46) | 49 (41) |
| Heart rate irregular | 0 (0) | 1 (1) | 1 (1) |
| Hemianopia | 1 (3) | 0 (0) | 1 (1) |
| Hemianopia homonymous | 1 (3) | 0 (0) | 1 (1) |
| Hemiparesis | 2 (5) | 4 (5) | 6 (5) |
| Herpes zoster | 0 (0) | 3 (4) | 3 (3) |
| Human chorionic gonadotropin increased | 0 (0) | 1 (1) | 1 (1) |
| Hyperbilirubinaemia | 0 (0) | 1 (1) | 1 (1) |
| Hyperglycaemia | 2 (5) | 2 (3) | 4 (3) |
| Hypertension | 5 (13) | 11 (14) | 16 (13) |
| Hyperthyroidism | 0 (0) | 1 (1) | 1 (1) |
| Hypoacusis | 1 (3) | 0 (0) | 1 (1) |
| Hypoaesthesia | 3 (8) | 3 (4) | 6 (5) |
| Hypoalbuminaemia | 0 (0) | 1 (1) | 1 (1) |
| Hypocalcaemia | 0 (0) | 1 (1) | 1 (1) |
| Hypokalaemia | 1 (3) | 1 (1) | 2 (2) |
| Hypophysitis | 0 (0) | 1 (1) | 1 (1) |
| Hypotension | 0 (0) | 1 (1) | 1 (1) |
| Hypothyroidism | 1 (3) | 2 (3) | 3 (3) |
| Immune-mediated hepatitis | 0 (0) | 1 (1) | 1 (1) |
| Immunisation reaction | 0 (0) | 1 (1) | 1 (1) |
| Incontinence | 0 (0) | 1 (1) | 1 (1) |
| Infection | 0 (0) | 1 (1) | 1 (1) |
| Influenza | 0 (0) | 1 (1) | 1 (1) |
| Infusion related reaction | 0 (0) | 1 (1) | 1 (1) |
| Ingrown hair | 0 (0) | 1 (1) | 1 (1) |
| Insomnia | 2 (5) | 2 (3) | 4 (3) |
| Itching scar | 0 (0) | 1 (1) | 1 (1) |
| Joint swelling | 2 (5) | 2 (3) | 4 (3) |
| Lacrimation increased | 1 (3) | 0 (0) | 1 (1) |
| Large intestine perforation | 0 (0) | 1 (1) | 1 (1) |
| Lethargy | 1 (3) | 1 (1) | 2 (2) |
| Lipase increased | 0 (0) | 3 (4) | 3 (3) |
| Lower respiratory tract infection | 2 (5) | 3 (4) | 5 (4) |
| Lung infection | 0 (0) | 1 (1) | 1 (1) |
| Lymphadenopathy | 0 (0) | 1 (1) | 1 (1) |
| Lymphocyte count decreased | 0 (0) | 4 (5) | 4 (3) |
| Lymphopenia | 3 (8) | 6 (8) | 9 (8) |
| Memory impairment | 1 (3) | 5 (6) | 6 (5) |
| Mobility decreased | 0 (0) | 3 (4) | 3 (3) |
| Mouth ulceration | 0 (0) | 2 (3) | 2 (2) |
| Mucosal inflammation | 0 (0) | 1 (1) | 1 (1) |
| Muscle spasms | 1 (3) | 4 (5) | 5 (4) |
| Muscle strain | 0 (0) | 1 (1) | 1 (1) |
| Muscle twitching | 0 (0) | 1 (1) | 1 (1) |
| Muscular weakness | 7 (18) | 10 (13) | 17 (14) |
| Musculoskeletal chest pain | 0 (0) | 2 (3) | 2 (2) |
| Musculoskeletal pain | 1 (3) | 0 (0) | 1 (1) |
| Musculoskeletal stiffness | 0 (0) | 1 (1) | 1 (1) |
| Myalgia | 0 (0) | 2 (3) | 2 (2) |
| Myopathy | 1 (3) | 2 (3) | 3 (3) |
| Nasopharyngitis | 3 (8) | 5 (6) | 8 (7) |
| Nausea | 11 (28) | 31 (39) | 42 (35) |
| Nephrolithiasis | 0 (0) | 1 (1) | 1 (1) |
| Neuralgia | 1 (3) | 1 (1) | 2 (2) |
| Neuropathy peripheral | 1 (3) | 0 (0) | 1 (1) |
| Neurotoxicity | 3 (8) | 2 (3) | 5 (4) |
| Neutropenia | 2 (5) | 4 (5) | 6 (5) |
| Neutrophil count decreased | 0 (0) | 4 (5) | 4 (3) |
| Oedema peripheral | 0 (0) | 2 (3) | 2 (2) |
| Onychomadesis | 0 (0) | 1 (1) | 1 (1) |
| Oral candidiasis | 2 (5) | 6 (8) | 8 (7) |
| Oral herpes | 0 (0) | 1 (1) | 1 (1) |
| Oropharyngeal pain | 2 (5) | 1 (1) | 3 (3) |
| Pain | 0 (0) | 2 (3) | 2 (2) |
| Pain in extremity | 3 (8) | 4 (5) | 7 (6) |
| Pain in jaw | 0 (0) | 1 (1) | 1 (1) |
| Pallor | 0 (0) | 1 (1) | 1 (1) |
| Palpitations | 0 (0) | 1 (1) | 1 (1) |
| Paraesthesia | 2 (5) | 2 (3) | 4 (3) |
| Partial seizures | 2 (5) | 1 (1) | 3 (3) |
| Periarthritis | 1 (3) | 0 (0) | 1 (1) |
| Periorbital cellulitis | 0 (0) | 1 (1) | 1 (1) |
| Peripheral swelling | 1 (3) | 1 (1) | 2 (2) |
| Platelet count decreased | 1 (3) | 5 (6) | 6 (5) |
| Pleural effusion | 0 (0) | 1 (1) | 1 (1) |
| Pollakiuria | 0 (0) | 3 (4) | 3 (3) |
| Poor quality sleep | 0 (0) | 1 (1) | 1 (1) |
| Presyncope | 0 (0) | 2 (3) | 2 (2) |
| Proctitis | 0 (0) | 1 (1) | 1 (1) |
| Productive cough | 0 (0) | 4 (5) | 4 (3) |
| Pruritus | 4 (10) | 31 (39) | 35 (29) |
| Pruritus generalised | 0 (0) | 1 (1) | 1 (1) |
| Pulmonary embolism | 3 (8) | 3 (4) | 6 (5) |
| Pyrexia | 1 (3) | 2 (3) | 3 (3) |
| Quadrantanopia | 0 (0) | 1 (1) | 1 (1) |
| Radiation skin injury | 2 (5) | 0 (0) | 2 (2) |
| Rash | 2 (5) | 24 (30) | 26 (22) |
| Rash generalised | 1 (3) | 2 (3) | 3 (3) |
| Rash macular | 0 (0) | 1 (1) | 1 (1) |
| Rash maculo-papular | 0 (0) | 8 (10) | 8 (7) |
| Rash papular | 0 (0) | 1 (1) | 1 (1) |
| Rash pruritic | 0 (0) | 3 (4) | 3 (3) |
| Raynaud's phenomenon | 0 (0) | 1 (1) | 1 (1) |
| Rectal haemorrhage | 0 (0) | 1 (1) | 1 (1) |
| Regurgitation | 0 (0) | 1 (1) | 1 (1) |
| Renal pain | 1 (3) | 1 (1) | 2 (2) |
| Respiratory tract infection | 0 (0) | 1 (1) | 1 (1) |
| Retinal disorder | 0 (0) | 1 (1) | 1 (1) |
| Rhinitis | 0 (0) | 1 (1) | 1 (1) |
| Rhinitis allergic | 0 (0) | 1 (1) | 1 (1) |
| Rhinorrhoea | 1 (3) | 0 (0) | 1 (1) |
| SARS-CoV-2 test positive | 1 (3) | 0 (0) | 1 (1) |
| Scar pain | 1 (3) | 0 (0) | 1 (1) |
| Seizure | 8 (20) | 21 (27) | 29 (24) |
| Sensory loss | 1 (3) | 0 (0) | 1 (1) |
| Septic rash | 0 (0) | 1 (1) | 1 (1) |
| Sinus tachycardia | 1 (3) | 0 (0) | 1 (1) |
| Sinusitis | 1 (3) | 1 (1) | 2 (2) |
| Skin injury | 0 (0) | 1 (1) | 1 (1) |
| Slow speech | 0 (0) | 1 (1) | 1 (1) |
| Somnolence | 1 (3) | 0 (0) | 1 (1) |
| Speech disorder | 0 (0) | 1 (1) | 1 (1) |
| Steroid diabetes | 1 (3) | 0 (0) | 1 (1) |
| Stomatitis | 1 (3) | 3 (4) | 4 (3) |
| Subcutaneous abscess | 1 (3) | 0 (0) | 1 (1) |
| Subdural haematoma | 0 (0) | 1 (1) | 1 (1) |
| Swelling face | 1 (3) | 1 (1) | 2 (2) |
| Swelling of eyelid | 0 (0) | 1 (1) | 1 (1) |
| Syncope | 1 (3) | 1 (1) | 2 (2) |
| Tension headache | 0 (0) | 1 (1) | 1 (1) |
| Terminal dribbling | 1 (3) | 0 (0) | 1 (1) |
| Thrombocytopenia | 5 (13) | 7 (9) | 12 (10) |
| Thyroid function test abnormal | 0 (0) | 1 (1) | 1 (1) |
| Tinnitus | 3 (8) | 3 (4) | 6 (5) |
| Tremor | 1 (3) | 0 (0) | 1 (1) |
| Unresponsive to stimuli | 1 (3) | 0 (0) | 1 (1) |
| Upper respiratory tract infection | 1 (3) | 4 (5) | 5 (4) |
| Upper-airway cough syndrome | 1 (3) | 0 (0) | 1 (1) |
| Urinary incontinence | 0 (0) | 1 (1) | 1 (1) |
| Urinary retention | 0 (0) | 1 (1) | 1 (1) |
| Urinary tract infection | 1 (3) | 3 (4) | 4 (3) |
| Urticaria | 0 (0) | 2 (3) | 2 (2) |
| Vestibular disorder | 0 (0) | 1 (1) | 1 (1) |
| Viral infection | 0 (0) | 1 (1) | 1 (1) |
| Vision blurred | 1 (3) | 2 (3) | 3 (3) |
| Visual impairment | 0 (0) | 1 (1) | 1 (1) |
| Vitamin D decreased | 0 (0) | 1 (1) | 1 (1) |
| Vitreous floaters | 0 (0) | 1 (1) | 1 (1) |
| Vomiting | 10 (25) | 18 (23) | 28 (24) |
| Weight decreased | 0 (0) | 4 (5) | 4 (3) |
| Weight increased | 1 (3) | 0 (0) | 1 (1) |
| White blood cell count decreased | 0 (0) | 1 (1) | 1 (1) |

**Supplemental Table 5: CTCAE grade 3 and above adverse events (MedDRA preferred term) by treatment group**

| **Adverse Event** | **Temozolomide alone**  ***n (%)*** | **Ipilimumab + Temozolomide**  ***n (%)*** | **Total**  ***n (%)*** |
| --- | --- | --- | --- |
| Abdominal pain | 0 (0) | 1 (1) | 1 (1) |
| Alopecia | 1 (3) | 0 (0) | 1 (1) |
| Anaemia | 0 (0) | 1 (1) | 1 (1) |
| Aphasia | 0 (0) | 1 (1) | 1 (1) |
| Ataxia | 1 (3) | 0 (0) | 1 (1) |
| Autoimmune colitis | 0 (0) | 4 (5) | 4 (3) |
| Brain oedema | 0 (0) | 1 (1) | 1 (1) |
| Cerebrospinal fluid leakage | 1 (3) | 0 (0) | 1 (1) |
| Colitis | 0 (0) | 2 (3) | 2 (2) |
| Confusional state | 2 (5) | 1 (1) | 3 (3) |
| COVID-19 | 0 (0) | 1 (1) | 1 (1) |
| COVID-19 pneumonia | 1 (3) | 0 (0) | 1 (1) |
| Decreased appetite | 0 (0) | 2 (3) | 2 (2) |
| Dehydration | 1 (3) | 1 (1) | 2 (2) |
| Diarrhoea | 0 (0) | 5 (6) | 5 (4) |
| Diarrhoea haemorrhagic | 0 (0) | 1 (1) | 1 (1) |
| Fatigue | 0 (0) | 2 (3) | 2 (2) |
| Hemianopia | 1 (3) | 0 (0) | 1 (1) |
| Hemiparesis | 1 (3) | 0 (0) | 1 (1) |
| Hyperglycaemia | 1 (3) | 0 (0) | 1 (1) |
| Hypertension | 1 (3) | 2 (3) | 3 (3) |
| Immune-mediated hepatitis | 0 (0) | 1 (1) | 1 (1) |
| Incontinence | 0 (0) | 1 (1) | 1 (1) |
| Large intestine perforation | 0 (0) | 1 (1) | 1 (1) |
| Lipase increased | 0 (0) | 1 (1) | 1 (1) |
| Lower respiratory tract infection | 0 (0) | 1 (1) | 1 (1) |
| Lung infection | 0 (0) | 1 (1) | 1 (1) |
| Lymphocyte count decreased | 0 (0) | 1 (1) | 1 (1) |
| Lymphopenia | 3 (8) | 2 (3) | 5 (4) |
| Mobility decreased | 0 (0) | 1 (1) | 1 (1) |
| Muscular weakness | 2 (5) | 3 (4) | 5 (4) |
| Neutropenia | 0 (0) | 2 (3) | 2 (2) |
| Neutrophil count decreased | 0 (0) | 3 (4) | 3 (3) |
| Periorbital cellulitis | 0 (0) | 1 (1) | 1 (1) |
| Platelet count decreased | 0 (0) | 2 (3) | 2 (2) |
| Presyncope | 0 (0) | 1 (1) | 1 (1) |
| Proctitis | 0 (0) | 1 (1) | 1 (1) |
| Pruritus | 1 (3) | 1 (1) | 2 (2) |
| Pulmonary embolism | 2 (5) | 3 (4) | 5 (4) |
| Pyrexia | 0 (0) | 2 (3) | 2 (2) |
| Rash | 0 (0) | 4 (5) | 4 (3) |
| Rash maculo-papular | 0 (0) | 2 (3) | 2 (2) |
| Seizure | 1 (3) | 7 (9) | 8 (7) |
| Slow speech | 0 (0) | 1 (1) | 1 (1) |
| Thrombocytopenia | 2 (5) | 6 (8) | 8 (7) |
| Unresponsive to stimuli | 1 (3) | 0 (0) | 1 (1) |
| Upper respiratory tract infection | 0 (0) | 1 (1) | 1 (1) |
| Urinary tract infection | 0 (0) | 1 (1) | 1 (1) |
| Vomiting | 3 (8) | 3 (4) | 6 (5) |
| Weight increased | 1 (3) | 0 (0) | 1 (1) |
| White blood cell count decreased | 0 (0) | 1 (1) | 1 (1) |
